# *Porphyromonas gingivalis* Gingipains Trigger a Proinflammatory Response in Human Monocyte-derived Macrophages Through the p38α Mitogen-activated Protein Kinase Signal Transduction Pathway 

**DOI:** 10.3390/toxins2030341

**Published:** 2010-03-10

**Authors:** Daniel Grenier, Shin-ichi Tanabe

**Affiliations:** Groupe de Recherche en Écologie Buccale, Faculté de Médecine Dentaire, Université Laval, 2420 de la Terrasse, Quebec City, Quebec, G1V 0A6, Canada; Email: Tanabes33238@aol.com

**Keywords:** *P. gingivalis*, proteinase, macrophage, cytokine, inflammation, p38α MAPK

## Abstract

*Porphyromonas gingivalis*, the major etiologic agent of chronic periodontitis, produces a broad spectrum of virulence factors, including Arg- and Lys-gingipain cysteine proteinases. In this study, we investigated the capacity of *P. gingivalis* gingipains to trigger a proinflammatory response in human monocyte-derived macrophages. Both Arg- and Lys-gingipain preparations induced the secretion of TNF-α and IL-8 by macrophages. Stimulation of macrophages with Arg-gingipain A/B preparation at the highest concentration was associated with lower amounts of cytokines detected, a phenomenon likely related to proteolytic degradation. The inflammatory response induced by gingipains was not dependent of their catalytic activity since heat-inactivated preparations were still effective. Stimulating macrophages with gingipain preparations was associated with increased levels of phosphorylated p38α MAPK suggesting its involvement in cell activation. In conclusion, our study brought clear evidence that *P. gingivalis* Arg- and Lys-gingipains may contribute to the host inflammatory response, a critical factor in periodontitis-associated tissue destruction.

## 1. Introduction

Periodontitis is an inflammatory disorder that results in the destruction of the supporting structures of the teeth, including the alveolar bone. It is the most important cause of tooth loss among adults and is initiated by an accumulation in subgingival sites of predominantly anaerobic Gram-negative bacteria called periodontopathogens. These bacteria and their products induce host cellular and humoral responses [1]. In most cases, these responses result in the elimination or control of the pathogens and significant periodontal disease does not become established or progress. However, in other cases, the continuous challenges of the host immune system by periodontopathogens initiate a number of host-mediated destructive processes [1]. 

Among the various bacterial species associated with the development of periodontitis, *Porphyromonas gingivalis* is suspected to be one of the most important causative agents of the chronic form of this disease [2]. *P. gingivalis* produces several virulence factors, including outer membrane vesicles, adhesins, lipopolysaccharides (LPS), hemolysins and proteinases [3,4]. Arg- and Lys-gingipain cysteine proteinases are the main endopeptidases produced by *P. gingivalis* and are both extracellular and cell-bound [5]. Two genes code for Arg-gingipains (*rgpA* and *rgpB*), and one codes for Lys-gingipain (*kgp*) [5,6]. RgpA and Kgp are made of a catalytic domain and a hemagglutinin/adhesion domain while RgpB does not possess the latter [6]. Because of their large activity spectrum, RgpA, RgpB, and Kgp are thought to play key roles in the pathogenesis of periodontitis. For instance, several studies have suggested that *P. gingivalis* gingipains can participate to tissue destruction directly by degrading host tissue proteins and indirectly by activating latent matrix metalloproteinases and inactivating host tissue inhibitors of metalloproteinases [7,8,9,10]. In addition to being critical in the pathogenic process, gingipains may play a number of physiological roles in bacteria, more particularly in controlling the expression of other virulence factors as well as in the stability and/or processing of extracellular or cell surface proteins [6].

Monocytes and macrophages, which are present in higher numbers in active periodontal lesions than in inactive sites [11], are key members of the innate immune system and play a critical role in the host response during chronic infections such as periodontitis [1]. Previous studies have shown the capacity of *P. gingivalis* cells to induce the secretion of proinflammatory cytokines by macrophages [12,13]. Cell surface LPS was identified as a major component contributing to the inflammatory response mediated by *P. gingivalis* [14]. In this study, we investigated the ability of *P. gingivalis* Arg- and Lys-gingipains to trigger a proinflammatory response in human macrophages. In addition, the signaling pathways leading to cytokine secretion were investigated.

## 2. Results

The Arg- and Lys-gingipain preparations were found to contain less than 5 pg/mL of contaminating LPS, indicating that trace endotoxins could not account for the macrophage responses observed. To investigate the gingipain-induced inflammatory response, monocyte-derived macrophages were stimulated for 18 h with the proteinase preparations (0.2, 1, and 5 units/mL). We first showed that treatments of macrophages with gingipains only slightly affected their viability. Compared to control cells, the viability never decreased by more than 9% (data not shown). 

**Figure 1:**
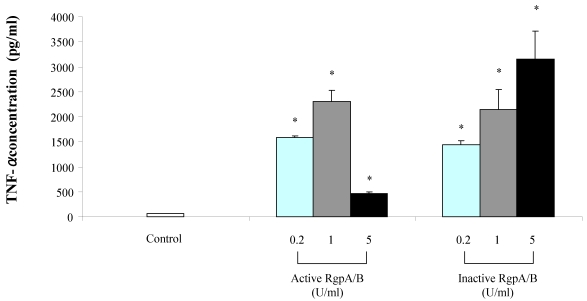
Secretion of TNF-α by macrophages stimulated with the Arg-gingipain A/B preparation (active and heat-inactivated). TNF-α concentrations were determined by ELISA and data are presented as means ± standard deviations of triplicate assays. Data were analyzed with the Student’s t-test (*: P < 0.05 *vs.* control).

**Figure 2:**
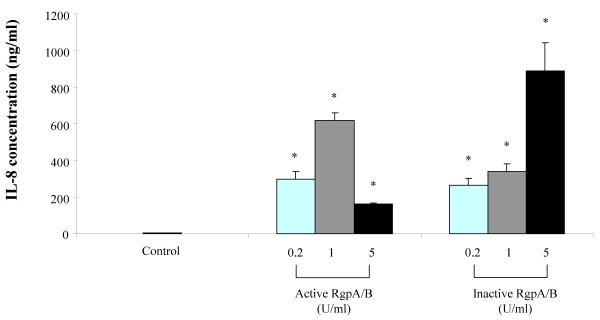
Secretion of IL-8 by macrophages stimulated with the Arg-gingipain A/B preparation (active and heat-inactivated). IL-8 concentrations were determined by ELISA and data are presented as means ± standard deviations of triplicate assays. Data were analyzed with the Student’s t-test (*: P < 0.05 *vs*. control).

Stimulating macrophages with the Arg-gingipain preparation significantly induced the secretion of TNF-α and IL-8 (Figure 1 and Figure 2). On the one hand, the amounts of TNF-α and IL-8 secreted were higher when stimulation was performed with active Arg-gingipains A/B at 0.2 and 1 unit/mL than at 5 units/mL. On the other hand, the secretion of TNF-α and Il-8 increased dose-dependently when macrophages were stimulated with the Arg-gingipain preparation inactivated by heat treatment. At 1 unit/mL, active Arg-gingipains A/B increased the secretion of TNF-α and IL-8 by 35 and 132 fold, respectively. At the same concentration, heat-inactivated Arg-gingipain A/B increased the secretion of TNF-α and IL-8 by 33 and 73 fold, respectively. To exclude the contribution of LPS contamination in cytokine release by macrophages, stimulations were performed in the presence of polymyxin B (10 µg/mL) in order to neutralize LPS. In all cases, the presence of polymyxin B had no significant effect on TNF-α and IL-8 secretion (data not shown).

**Figure 3:**
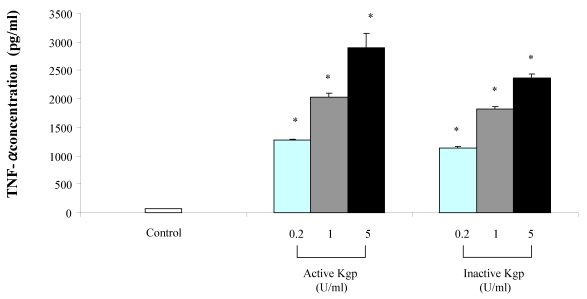
Secretion of TNF-α by macrophages stimulated with the Lys-gingipain preparation (active and heat-inactivated). TNF-α concentrations were determined by ELISA and data are presented as means ± standard deviations of triplicate assays. Data were analyzed with the Student’s t-test (*: P < 0.05 *vs.* control).

**Figure 4:**
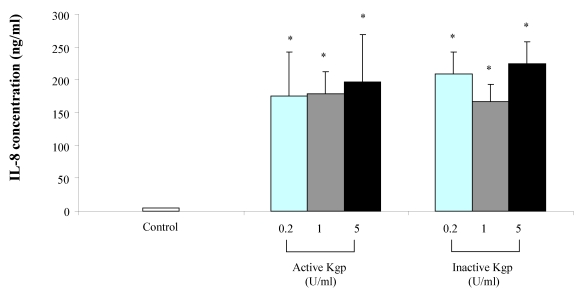
Secretion of IL-8 by macrophages stimulated with the Lys-gingipain preparation (active and heat-inactivated). IL-8 concentrations were determined by ELISA and data are presented as means ± standard deviations of triplicate assays. Data were analyzed with the Student’s t-test (*: P < 0.05 *vs.* control).

Macrophage stimulation with the Lys-gingipain preparation, either active or heat-inactivated, dose-dependently increased TNF-α secretion (Figure 3). Regarding IL-8, the Lys-gingipain preparation induced its secretion by macrophages although it was not dose-dependent (Figure 4). Similar results were obtained when the Lys-gingipain preparation was heat-treated. At 1 unit/mL, active Lys-gingipain increased the secretion of TNF-α and IL-8 by 31 and 38 fold, respectively. As for the Arg-gingipain, the presence of polymyxin B did not modify the amounts of secreted cytokines following stimulation of macrophages with the Lys-gingipain preparation (data not shown).

Changes induced by the gingipain preparations in the amount of phosphorylated macrophage intracellular kinases known to be involved in inflammatory signaling pathways were characterized by a multiplex ELISA analysis. As reported in Table 1, both the Arg- and Lys-gingipain preparations induced increases in phosphorylated p38α MAPK (+343% for RgpA/B and +227% for Kgp) and to a lesser extent ERK1/2 (+25% for RgpA/B and +32% for Kgp). The amounts of phosphorylated JNK2 were not significantly affected. Since p38α MAPK appeared to be the most important kinase involved in cell activation by gingipains, we used the highly specific inhibitor SB203580 to confirm its role (Table 2). In the presence of SB203580, the RgpA/B- or Kgp-induced secretion of TNF-α was reduced by 87% and 79% respectively while that of IL-8 was decreased by 92% and 69% respectively. 

**Table 1:** Effects of Arg- and Lys-gingipain treatments in the amounts of phosphorylated intracellular kinases in macrophages. Macrophages were treated for 90 min with the gingipain preparations. Cell lysates were prepared as described in Materials and Methods and assayed by the Searchlight Kinase Service (Pierce Biotechnology).

**Protein**	**Signal (% Change)**		
**Full name and abbreviation**	**Epitope**	**Unstimulated *vs.* RgpA/B**	**Unstimulated *vs.* Kgp**
Extracellular regulated protein-serine kinase 1/2 (p42, p44 MAP kinase); ERK1/2	T202/Y204	+25	+32
Jun N-terminus protein-serine kinase (stress-activated protein kinase 2); JNK2	T183/Y185	+5	0
Mitogen-activated protein-serine kinase p38 alpha; p38α MAPK	T180/Y182	+343	+227

**Table 2:** Effect of SB203580, a specific inhibitor of p38α MAPK, on secretion of TNF-α and IL-8 by gingipain-stimulated macrophages.

**Gingipain preparation (1 unit/mL)**	**% inhibition in the presence of SB203580**	
	**TNF-**α	**IL-8**
RgpA/B	87%	92%
Kgp	79%	69%

## 3. Discussion

Arg- and Lys-gingipains, the major endopeptidases produced by *P. gingivalis*, are thought to be critical virulence factors since deficient mutants are less pathogenic in animal models [15,16]. In addition to degrade key tissue and plasma proteins [5], gingipains may contribute to the development and maintenance of an inflammatory state. A first mechanism that has been proposed is through activation of the kallikrein/kinin-generating cascade, which results in increased vascular permeability [17]. A second mechanism involves activation by Arg-gingipains of blood coagulation factor X to generate activated factor X (FXa) [18], which can modulate the production of proinflammatory cytokines [19]. More recently, it has been reported that *P. gingivalis* gingipains can activate different cell types leading to the secretion of inflammatory mediators [20,21,22]. 

In this study, we investigated the capacity of *P. gingivalis* gingipains to induce an inflammatory response in macrophages through activation of intracellular kinases. We showed that both the Arg- and Lys-gingipain preparations induced the production of TNF-α and IL-8 by macrophages. Two different results support that contaminant LPS is not involved in macrophage activation. First, a chromogenic *Limulus* amoebocyte lysate assay performed on gingipain preparations ruled out the possibility thatcontaminant LPS accounted for the responses observed. Second, the presence of polymyxin B, an LPS inhibitor universally used, did not modify macrophage activation by gingipains. When used at 1 unit/mL, the Arg-gingipain preparation induced the secretion of larger amounts of the cytokines in comparison with the Lys-gingipain preparation. Given the biological properties associated with TNF-α and IL-8, they are likely to contribute to periodontitis [23,24]. TNF-α can induce the expression of adhesion molecules and mediators that facilitate and amplify the inflammatory response, matrix metalloproteinase production and bone resorption [23]. IL-8 is a potent chemotactic and activating cytokine produced by several cell types, including macrophages. It amplifies immune responses by attracting and inducing immune cells such as polymorphonuclear leukocytes, monocytes, and macrophages to produce pro-inflammatory mediators. The stimulation of chemokine production by periodontopathogens is believed to play an important role in initiating inflammatory reactions in gingival tissues [24]. IL-8 is also able to induce differentiation of hematopoietic precursors of the monocyte-macrophage lineage into osteoclasts suggesting an important role in bone resorption [25]. IL-8 concentrations in the gingival crevicular fluid of inflamed periodontal sites correlate with the severity of periodontitis [26]. Stashenko *et al.* [27] reported a highly significant correlation between the levels of TNF-α and IL-8 in the periodontal tissues of diseased sites, suggesting that the expression of these two mediators is coordinated.

In a recent study, Uehara *et al.* [21] demonstrated that Arg- and Lys-gingipains of *P. gingivalis* stimulate the secretion of proinflammatory cytokines by monocytes through protease-activated receptor (PAR)-1, -2, and -3 with synergistic effects when used in combination with pathogen-associated molecular patterns. This capacity was dependent on the proteolytic cleavage of PARs by gingipains. In our study, a different mechanism of activation was involved since stimulation of macrophages leading to TNF-α and IL-8 secretion occurred with heat-inactivated gingipains. A similar observation was reported by Fitzpatrick *et al*. [22] who observed that gingipain-induced IL-1β secretion by differentiated macrophages involves a nonproteolytic mechanism.

It was found that stimulating macrophages with the highest concentration (5 units/mL) of Arg-gingipain resulted in lower amounts of both TNF-α and IL-8 detected in cell-free culture supernatant when compared with stimulation with 0.2 and 1 unit/mL. This does not result from loss of cell viability and consequently reduced secretion of cytokines since gingipain preparations showed no significant cytotoxicity. This decreased amount of cytokines is most likely related to proteolytic degradation. Indeed, *P. gingivalis* gingipains have been reported to degrade TNF-α [28] and IL-8 [29]. The biological significance of such degradation may be important during the course of periodontitis. Indeed, proteolysis of cytokines by *P. gingivalis* may deregulate the complex host cytokine network responsible for periodontal health and the maintenance of both innate and acquired immunity. The cytokine degradation was gingipain dose-dependent. It suggests that degradation of cytokines may occur especially near the bacterial plaque, where gingipain concentrations are high. In gingival tissue, where bacterial and gingipain concentrations are low, the degradation of cytokines may be a minor phenomenon allowing the development of an intense inflammatory reaction in response to periodontopathogen stimulations.

Upon receptor activation by stimuli, signaling cascades are activated to lead to inflammatory mediator secretion. In our study, the *P. gingivalis* gingipains appeared to activate the p38α MAPK signal transduction pathway. The p38 MAPK is well known to play a key role in inflammatory and chemokine regulation at both the transcriptional and the post-transcriptional levels [30]. Evidences have been presented to support an important role of p38 MAPK signaling in periodontal disease [31].

## 4. Experimental Section

### 4.1. Bacterial strains and growth conditions

*P. gingivalis* ATCC 33277 and two derivative mutants (KDP112 [*rgpA*, *rgpB*] and KDP129 [*kgp*]) were grown in Todd Hewitt broth (THB, BBL Microbiology Systems, Cockeysville, MD) supplemented with hemin (10 µg/mL) and vitamin K (1 µg/mL). The mutants, which were kindly provided by K. Nakayama (Nagasaki University, Japan), were constructed by insertional mutations as described previously [32,33]. All cultures were incubated in an anaerobic chamber (N_2_-CO_2_-H_2_, 80:10:10) at 37 °C. 

### 4.2. Proteinase purification

Arg-gingipains A/B and Lys-gingipain were purified from *P. gingivalis* KDP129 and KDP112, respectively. The use of these mutants facilitated the purification of the gingipains, which are difficult to separate from one another. Gingipains were purified from cell envelope extracts by affinity chromatography on arginine Sepharose 4B (Amersham Pharmacia Biotech, Baie d’Urfé, QC), as previously described [34,35]. The purity of the preparations was assessed by SDS-PAGE analysis and silver staining. One unit of enzyme activity was defined as the amount required to release 1 nM of *p*-nitroaniline per h using the chromogenic synthetic substrates benzoyl-Arg-*p*-nitroanilide for Arg-gingipain activity and N-*p*-tosyl-Gly-Pro-Lys-*p*-nitroanilide for Lys-gingipain activity. The presence of contaminant LPS in the gingipain preparations was determined using a quantitative chromogenic *Limulus* amoebocyte lysate assay according to the manufacturer's directions (BioWhittaker, Walkersville, MD).

### 4.3. Preparation and stimulation of monocyte-derived macrophages

U937 cells, a macrophage-like cell line, were obtained from the American Type Culture Collection (ATCC; Rockville, MD) and grown in RPMI 1640 medium supplemented with penicillin (50 µg/mL), streptomycin (50 µg/mL) and 10% heat-inactivated fetal bovine serum (FBS) at 37 °C in a 5% CO_2 _atmosphere. The cells were seeded at 2 × 10^5^ cells/mL and split approximately three times per week to maintain the cell concentration between 1 × 10^5^ and 2 × 10^6^ viable cells/mL for optimal growth. To differentiate monocytes into adherent macrophages, phorbol-12-myristate 13-acetate (PMA; Sigma Chemical Co., St. Louis, MO) was added at a concentration of 1 ng/mL and the cells were cultivated for 48 h. Thereafter, the cells were incubated in PMA-free medium for 1 d prior to use. Macrophages were treated with gingipain preparations (0.2, 1 and 5 units/mL) for 18 h at 37 °C in a 5% CO_2 _atmosphere. To investigate whether the catalytic activity of gingipains was involved in cell activation, proteinase preparations were treated at 56 °C for 30 min prior to stimulate macrophages. Such a treatment completely abolished the hydrolytic activity of gingipains. To ensure that contaminating LPS were not responsible for macrophage stimulation, treatments were also performed in the presence of polymyxin B at a final concentration of 10 µg/mL. All assays were run in triplicate in two separate experiments.

### 4.4. Macrophage viability

An MTT (3-[4,5-diethylthiazol-2-yl]-2,5-diphenyltetrazolium bromide) test performed according to the manufacturer’s protocol (Roche Diagnostics, Mannheim, Germany) was used to test the viability of macrophages following treatment with Arg- and Lys-gingipain preparations.

### 4.5. Determination of TNF- and IL-8 secretion

Commercial enzyme-linked immunosorbent assay (ELISA) kits (R&D Systems, Minneapolis, MN, USA) were used to quantify TNF-α and IL-8 concentrations in the cell-free culture supernatants according to the manufacturer’s protocol. The absorbance at 450 nm was read using a microplate reader with the wavelength correction set at 550 nm. The rated sensitivities of the commercial ELISA kits were 15.6 pg/mL for TNF-α and 31.2 pg/mL for IL-8. The differences between the means were analyzed for statistical significance at P < 0.05 using the Student’s t-test.

### 4.6. Analysis of phosphorylated macrophage intracellular kinases

Searchlight kinase assays, which provide quantitative information on phosphorylated intracellular protein kinases, were performed by Pierce Biotechnology (Woburn, MA, USA). The multiplex sandwich ELISA assays use highly specific immunological reagents and a sensitive chemiluminescent detection system. Macrophages were treated for 90 min with the gingipain preparations (1 unit/mL). The cells were washed twice with ice-cold 50 mM phosphate-buffered saline (pH 7.2) and homogenized at 4 °C in a buffer containing 25 mM Tris-HCl (pH 7.6), 150 mM sodium chloride, 1% Nonidet P-40, 1% sodium deoxycholate, and 1% sodium dodecyl sulfate. The buffer was supplemented with Halt Protease inhibitor cocktail and Halt Phosphatase inhibitor cocktail (Pierce Biotechnology), which was used according to the manufacturer’s instructions. The cells were broken by sonication on ice and the lysates were centrifuged at 13,000× *g for 10 min at* 4 °C to remove cell debris. Protein concentrations in the supernatant fractions were determined using the Bradford assay (Bio-Rad Laboratories, Ltd., Mississauga, ON, Canada) and adjusted to 1 mg/mL. Concentrations of phosphorylated intracellular kinases in the samples were measured in duplicate at three dilutions and the mean values of the best measurements were calculated. Data reported in the manuscript are representative of the two experiments.

### 4.7. Effect of a p38 MAPK inhibitor on macrophage activation by gingipains

A specific inhibitor of p38 MAPK, SB203580 purchased from Calbiochem (Mississauga, ON, Canada), was added (2 µM) to macrophages 2 h prior to being stimulated with gingipain preparations (1 unit/mL) for 24 h. TNF-α and IL-8 concentrations in the cell-free culture supernatants were quantified by ELISA as described above.

## 5. Conclusions

The host inflammatory response is a critical factor in periodontitis-associated tissue destruction. Both Arg- and Lys-gingipains produced by *P. gingivalis* induced the secretion of TNF-α and IL-8 by macrophages. This inflammatory response involves the p38α MAPK signal transduction pathway.
